# Effectiveness of diclofenac versus acetaminophen in primary care patients with knee osteoarthritis: [NTR1485], DIPA-Trial: design of a randomized clinical trial

**DOI:** 10.1186/1471-2474-11-7

**Published:** 2010-01-12

**Authors:** Saskia PJ Verkleij, Pim AJ Luijsterburg, Bart W Koes, Arthur M Bohnen, Sita MA Bierma-Zeinstra

**Affiliations:** 1 Department of General Practice, Erasmus MC, University Medical Center Rotterdam, Netherlands

## Abstract

**Background:**

Osteoarthritis is the most frequent chronic joint disease which causes pain and disability of especially hip and knee. According to international guidelines and the Dutch general practitioners guidelines for non-traumatic knee symptoms, acetaminophen should be the pain medication of first choice for osteoarthritis. However, of all prescribed pain medication in general practice, 90% consists of non-steroidal anti-inflammatory drugs compared to 10% of acetaminophen. Because general practitioners may lack evidence showing a similar efficacy of acetaminophen and non-steroidal anti-inflammatory drugs, we present the design of a randomized open-label trial to investigate the efficacy of a non-steroidal anti-inflammatory drug (diclofenac) compared with acetaminophen in new consulters with knee osteoarthritis in general practice.

**Methods/Design:**

Patients aged 45 years or older consulting their general practitioner with non-traumatic knee pain, meeting the clinical American College of Rheumatology criteria, and with a pain severity score of 2 or higher (on a 0-10 scale), will be randomly allocated to either diclofenac (maximum daily dose of 150 mg) or acetaminophen (maximum daily dose of 3000 mg) for 2 weeks and, if required, an additional 1-2 weeks, with a total follow-up period of 12 weeks. The primary outcomes are knee pain measured with a daily diary, and pain and function measured with the Knee Injury and Osteoarthritis Outcome Score (KOOS) at baseline, and at 3, 6, 9, and 12-weeks follow-up. Secondary outcomes are patients' perceived recovery, quality of life, medical, patient, and productivity costs, compliance to therapy, co-interventions, and adverse reactions.

**Discussion:**

The successful completion of this trial would lead to a better understanding of which medication should be used in the treatment of primary care patients with mild knee osteoarthritis.

**Trial registration:**

Dutch trial registry NTR1485.

## Background

Osteoarthritis (OA) is the most frequent chronic joint disease causing pain and disability of especially hip and knee [1]. For most patients the general practitioner (GP) is the initial caregiver and may provide advice and/or pain medication. International guidelines and the Dutch GP guidelines for treating non-traumatic knee symptoms recommend acetaminophen as medication of first choice in the management of OA pain [2-4]. However, a prospective cohort of first consulters with non-traumatic knee symptoms in 40 Dutch general practices showed that GPs prescribed pain medication in 27% of these patients, 90% received non-steroidal anti-inflammatory drugs (NSAIDs) and only 10% received acetaminophen (Belo JN, Berger MY, Koes BW, Bierma-Zeinstra SMA: Medical treatment and medical consumption in adults with nontraumatic knee complaints in general practice. Submitted).

Despite general consensus that acetaminophen has a better safety profile, there may be insufficient evidence for the efficacy of acetaminophen in mild OA to convince GPs that NSAIDs should be avoided as first choice medication. Indeed, a systematic review of 15 randomized clinical trials (RCTs; median length 6 weeks) on the comparative effectiveness of NSAIDs versus acetaminophen in patients with hip/knee OA reported that although acetaminophen was more effective than placebo, it provided less pain relief than NSAIDs [5]. The efficacy of NSAIDs was especially found in patients with moderate to severe OA, whereas others report that the efficacy of NSAIDs and acetaminophen is probably similar in patients with mild OA [6].

A limitation of most RCTs is that they seldom include patients consulting for OA (i.e. new patients) but mostly prevalent cases already receiving treatment for OA. Most studies included a highly selected patient group already using a daily dose of NSAIDs and needing a wash-out period prior to randomization [7-10]. One trial reported (not surprisingly) that prior use of NSAIDs predicted a better response of NSAIDs compared to acetaminophen [8]. Therefore, these latter studies do not represent patients with OA in general practice, or patients who consult their GP for the first time with a new episode of complaints.

In view of the lack of trials comparing the efficacy of NSAIDs with acetaminophen in new consulters with OA, we designed an RCT to explore whether there is a clinically relevant difference between diclofenac (an NSAID) and acetaminophen in new patients with knee OA in general practice. A pragmatic open-label design was chosen to approximate GPs' daily practice and because patients are aware of the type of prescribed medication. Secondary aims were to establish: 1) whether there are predefined predictors of treatment responders after 4-6 weeks and at 12-weeks follow-up, and 2) the cost-effectiveness of diclofenac compared to acetaminophen in patients with knee OA in primary care over a 12-week period.

Presented below is the protocol of the diclofenac versus acetaminophen trial (DIPA trial), which is registered in the Dutch trial registry (NTR1485) [11].

## Methods/Design

### Study design

This study is a pragmatic randomized open-label trial with a follow-up period of 12 weeks. In this design, the patients, researchers and GPs are not blinded for the assigned treatment. The study is approved by the Medical Ethics Committee of the Erasmus Medical Center (MC).

**Figure 1:**
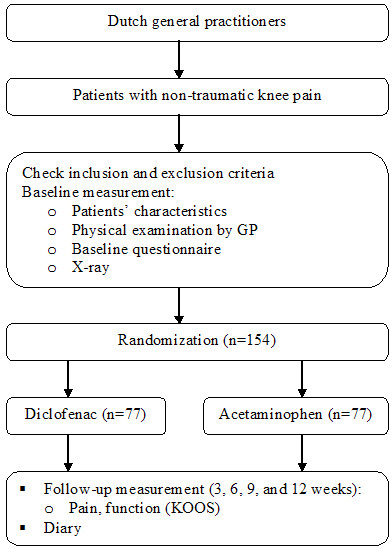
**Flow chart of the study**.

Figure 1 presents the flowchart of the study.

### Inclusion/exclusion criteria

Patients are eligible for the DIPA trial (Table 1) if they meet all four inclusion criteria: 1) consulting their GP for a new episode of non-traumatic knee pain. A new episode of knee pain is defined as pain presented to the GP for the first time, or if a patient did not consult the GP with these symptoms in the previous 3 months [12], 2) aged 45 years or older, 3) meeting the clinical American College of Rheumatology (ACR) criteria for OA of the knee [13], and 4) having a pain severity of 2 or more (on a 0-10 scale).

**Table 1:** Inclusion and exclusion criteria of the DIPA trial.

Inclusion Criteria	Exclusion Criteria
People with a new episode of non-traumatic knee pain	Contra-indication for NSAID or acetaminophen
Age ≥ 45 years	Arthroplasty/osteotomy
Comply with the clinical ACR criteria*	Already on NSAID or acetaminophen use^
Pain severity scale ≥ 2 on a 11-point numeric rating scale	Surgery or major trauma of affected knee in previous 6 months
	Oral corticosteroid use
	Myocardial infarction or stroke in previous 6 months

*Clinical ACR criteria: Age > 50 years, stiffness < 30 minutes, crepitus, bony tenderness, bony enlargement, and no palpable warmth. Patients comply with the clinical ACR criteria if they meet at least 3 of the 6 criteria.

^Excluded are those with a pre-study medication use comparable with the study dose of diclofenac (≥ 150 mg/day) or acetaminophen (≥ 3000 mg/day)

Patients are excluded if they are: 1) contra-indicated for NSAID or acetaminophen use, i.e. gastrointestinal bleedings in history or active, blood dyscrasia, bone marrow depression (myelosuppression), serious heart failure, serious liver or kidney disease (glomerular filtration < 30 ml/min), alcoholism, colitis ulcerosa, Crohn's disease, sulphite hypersensitivity, asthma, urticaria, angioedema, nasal polyps or rhinitis after use of acetylsalicylic acid or other prostaglandin synthetase inhibitors, or use of anti-depressive medication (SSRIs), 2) having an arthroplasty or osteotomy of the knee on the contralateral or unilateral side, 3) already taking NSAIDs or acetaminophen at doses similar to or higher than the study dose, 4) surgery or major trauma of the affected joint within the previous 6 months, 5) myocardial infarction or stroke in the last 6 months, and 6) oral use of a corticosteroid.

### Patient selection

An academic research network of GPs in the south-west of the Netherlands agreed to participate and to refer patients who consult for a new episode of non-traumatic knee pain to the DIPA trial. The GP takes the patient's history and performs the physical examination as part of the usual daily care. The GP gives study information to the patient, and sends the patient's name and information regarding history taking/physical examination by fax to the research department at Erasmus MC. Within two days after the GP visit, patients are contacted (by the researcher), checked for eligibility (in- and exclusion criteria), and asked for written informed consent. Baseline measurements and randomization then take place.

### Randomization

Patients are allocated to the diclofenac or the acetaminophen group using a randomization list (with random blocks of 4, 6 or 8) produced by a computer-generated table. The GP is informed about the randomization results and sends a prescription of the allocated medication to the patient's pharmacy.

### Interventions

Patients are randomly allocated to either diclofenac (maximum daily intake of 3 × 50 mg) or acetaminophen (maximum daily intake of 3 × 1000 mg). Both medications are prescribed in accordance with the Dutch clinical guidelines for GPs for non-traumatic knee symptoms [2]. The guideline recommends analgesics for 2 weeks and, if required, for an additional 1-2 weeks [2]. This is in accordance with the EULAR and OARSI recommendations [3,4]. Patients in the diclofenac group with an increased risk of gastro-intestinal problems will also receive a mucosal protector (e.g. omeprazol once daily, 20 mg). Patients at increased risk of gastro-intestinal problems are 60 years or older and/or have a serious co-morbidity (e.g. rheumatic disease and diabetes mellitus). Patients take their allocated medication on demand, and can change their medication intake when their pain level alters. This leads to an approach that is close to usual daily care.

### Outcome measures

The primary outcomes of this study are: 1) pain and function measured with the Knee Injury and Osteoarthritis Outcome Score (KOOS) [14] and 2) pain assessed with an 11-point numeric rating scale (NRS) in a diary [15]. Secondary outcomes are: 1) patients' perceived pain measured every 3 weeks on the 11-point NRS [15], 2) patients' perceived recovery measured on a 7-point Likert scale (1 = completely recovered; 7 = worse than ever), 3) constant and intermittent pain measured with the Intermittent and Constant Osteoarthritis Pain (ICOAP) questionnaire [16], 4) patients' quality of life assessed with the EuroQol instrument EQ-5D [17], 5) all direct medical, patient and productivity costs measured with the PROductivity and DISease Questionnaire (PRODISQ) [18], 6) compliance to therapy assessed in the diary, 7) co-interventions (e.g. changes in doses of co-medication), and 8) adverse reactions.

### Questionnaires

The primary and secondary outcome measurements are assessed with questionnaires and diaries. During the study, patients fill out a total of 5 questionnaires (at baseline and at 3, 6, 9, and 12-weeks follow-up). After the informed consent and before randomization, the patient fills out the baseline questionnaire. After the baseline questionnaire, patients receive a follow-up questionnaire every 3 weeks.

Five validated instruments are used in all 5 questionnaires.

1) The KOOS measures the functional status of patients with knee OA [14]. The KOOS consists of 5 subscales: pain, symptoms, activities of daily living, sport and function, and knee-related quality of life. The Dutch version of the KOOS is validated and suitable for use in patients with mild and moderate OA [14]. The KOOS questionnaire is an extension of the Western Ontario and McMaster osteoarthritis index (WOMAC), and WOMAC scores of pain and function can be calculated from the KOOS [19-21]. The WOMAC is recommended for use in elderly subjects with knee OA [19].

2) The measure of Intermittent and Constant Osteoarthritis Pain (ICOAP) identifies different types of pain due to OA. The ICOAP is a reliable and valid to measure constant and intermittent pain [16].

3) The 11-point NRS measures the perceived level of pain intensity (0 = no pain; 10 = worst pain ever) [22-25]. The NRS is a valid measurement to score pain intensity level [22].

4) The EuroQol (EQ-5D) measures quality of life. The EuroQol is a generic questionnaire and consists of 5 dimensions: mobility, self-care, usual activities, pain/discomfort, and anxiety/depression [17]. The EuroQol allows to evaluate the cost-effectiveness of a healthcare intervention [26,27] and can be converted into utilities to calculate Quality Adjusted Life Years (QALYs) [28].

5) The PRODISQ measures all direct medical, patient, and productivity costs. The PRODISQ consists of 7 modules. In the present study, only modules 1-5 are used because these questions are related to the individual patient, whereas modules 6 and 7 are utilized by management. Modules 1-5 cover: 1) demography and disease, 2) profession, working situation, and income, 3) absence from work, 4) compensation mechanisms, and 5) productivity costs whilst at work (efficiency loss) [18].

Besides these validated questionnaires, the baseline questionnaire addresses patient characteristics (age, gender, weight, height, and social status), knee-related characteristics (history and localisation of knee symptoms), problems at work due to knee problems, and co-morbidities. The four follow-up questionnaires measure medication use, adverse reactions, medical consumption, patients' perceived recovery, and knee-related characteristics.

Table 2 presents an overview of the questionnaire items.

**Table 2:** Overview of questionnaire items.

	0 weeks **B.Q**.	3 weeks **F.U.Q**.	6 weeks **F.U.Q**.	9 weeks **F.U.Q**.	12 weeks **F.Q**.	Diary
**Demographics**						
Age, gender, weight, height, and social status	**X**					
**Outcome measures**						
Pain score (NRS)	**X**	**X**	**X**	**X**	**X**	**X**
Pain score (KOOS)	**X**	**X**	**X**	**X**	**X**	
Function score (KOOS)	**X**	**X**	**X**	**X**	**X**	
Perceived recovery	**X**	**X**	**X**	**X**	**X**	
Constant pain, and pain that comes and goes (ICOAP)	**X**	**X**	**X**	**X**	**X**	
Quality of life (EuroQol)	**X**	**X**	**X**	**X**	**X**	
Direct medical, patient, and productivity costs (PRODISQ)					**X**	
Compliance						**X**
Adverse reactions		**X**	**X**	**X**	**X**	
**Other outcomes**						
Knee-related characteristics (History, duration, and localisation)	**X**					
Co-morbidities	**X**					
Medication use		**X**	**X**	**X**	**X**	**X**
Medical consumption (Visit to GP, medical specialist, physical therapist, etc.)		**X**	**X**	**X**	**X**	

B.Q. = Baseline Questionnaire; F.U.Q. = Follow-up Questionnaire; F.Q. = Final Questionnaire; KOOS = Knee Osteoarthritis Outcome Score; NRS = Numeric Rating Scale; ICOAP = Intermittent and Constant OsteoArthritis Pain; PRODISQ = PROductivity and DISease Questionnaire

### Pain diary

During the DIPA trial, patients fill out a diary to score daily pain (using an 11-point NRS), medication use, and compliance. Being a pragmatic trial, patients may change their medication dosage when pain alters. These alterations may be important for interpreting the results of the trial. Therefore, information on compliance to the allocated treatment is also collected.

### Sample size

The sample size is calculated to detect clinically relevant differences in pain and function between the two groups (diclofenac versus acetaminophen), measured by the KOOS during the 12-week study period. To detect a clinically relevant difference of 10 points (15%) on the KOOS pain score between the two treatment groups after 12 weeks, 73 patients per group are needed (power 95%, alpha 0.05, one-sided testing). Based on an expected 5% loss to follow, 154 patients (2 × 77) should be included.

### Statistical analyses

All analyses will be performed on an intention-to-treat basis, analyzing all patients in the treatment group to which they were randomly allocated. Analysis per protocol will also be conducted, analyzing only those patients that have measures on the primary outcome measurement at both baseline and 12-weeks follow-up. Descriptive data of baseline characteristics will be presented for both groups to check comparability. Generalized estimating equation (GEE) analysis will be conducted to investigate (longitudinally) the 2, 4, and 6 weeks effectiveness of diclofenac compared to acetaminophen for pain assessed with the diary. Differences between the two groups over the 12-week follow-up will also be assessed with GEE. The outcome variables are pain (measured with the NRS), and pain and function (assessed with the KOOS). Using GEE, the correlation of multiple measurements within one patient is taken into account [29].

To detect predictive variables for treatment responders at 12-weeks follow-up multivariate regression analyses will be used. Treatment response is defined based on the OMERACT-OARSI responder criteria [30,31] as a high improvement in pain or function of ≥ 50%, or an improvement on pain ≥ 20%, and/or function ≥ 20%.

In addition, a cost-utility analysis will be performed that expresses health improvements in QALYs assessed with the EuroQol. If the course of OA (and its related costs) appears to fluctuate (particularly if the difference between treatment arms is not stable over time), an additional modeling study using a Markov model will be performed. Statistical methods will be used to describe uncertainty in costs and effects estimates based on patient data. A 95% confidence interval for the cost-utility ratio will be calculated and an acceptability curve presented. In case of a modeling study, a probabilistic sensitivity analysis will be performed.

## Discussion

Recruitment of the 154 patients has started and will end in 2010. We expect to report study results in 2011. The successful completion of this trial would lead to a better understanding of which medication should be used in the treatment of primary care patients with mild knee osteoarthritis.

## Competing interests

The authors declare that they have no competing interests.

## Authors' contributions

SMABZ, BWK, and PAJL conceived the study, developed the trial design, and contributed to writing the article. AMB contributed to the trial design and to writing the article. SPJV is the coordinator, participated in the design of the study, and prepared the article. All authors have read and approved the final version of the article.

## Pre-publication history

The pre-publication history for this paper can be accessed here:

http://www.biomedcentral.com/1471-2474/11/7/prepub
